# Effectiveness of neural mobilization on pain intensity, disability, and physical performance in adults with musculoskeletal pain—A protocol for a systematic review of randomized and quasi-randomized controlled trials and planned meta-analysis

**DOI:** 10.1371/journal.pone.0264230

**Published:** 2022-03-10

**Authors:** Frederico Mesquita Baptista, Eduardo Brazete Cruz, Vera Afreixo, Anabela G. Silva

**Affiliations:** 1 Department of Medical Sciences, University of Aveiro, Aveiro, Portugal; 2 Department of Physiotherapy, Escola Superior de Saúde, Instituto Politécnico de Setúbal, Setúbal, Portugal; 3 CIDMA–Center for Research and Development in Mathematics and Applications, Department of Mathematics, University of Aveiro (UA), Aveiro, Portugal; 4 Center for Health Technology and Services Research (CINTESIS.UA), School of Health Sciences, University of Aveiro, Aveiro, Portugal; Illawarra Shoalhaven Local Health District, AUSTRALIA

## Abstract

Recent studies show that musculoskeletal conditions contribute significantly to years lived with disability considering the entire global population. Pain and functional disability are the main problems that people with these conditions suffer. Neural mobilization has been shown to be an effective intervention in the treatment of musculoskeletal pain within individual trials, also contributing to improved functionality. Some systematic reviews have been carried out during the last years with the aim of synthesizing the scientific evidence on the use of neural mobilization techniques in the treatment of musculoskeletal disorders. However, they varied a lot in the methodological approaches and, consequently, in the findings and conclusions. Thus, this document is a planned protocol of a comprehensive systematic review with meta-analysis that we intend to carry out to review the scientific literature regarding up-to-date evidence on the use of neural mobilization in the management of people suffering from musculoskeletal pain disorders. The study designs that we will consider as inclusion criteria will be randomized and quasi-randomized clinical trials. The target population will be adults and older adults with musculoskeletal pain. Any controlled trial using any neural mobilization technique as an intervention in one of the trial groups will be included. The main outcomes of interest will be pain, functional status, and physical performance tests (muscle strength, flexibility, and balance). There will be no restrictions on follow-up time or type of setting. The risk of bias of the included studies will be assessed by the RoB 2 tool and the certainty of the evidence will be evaluated using the comprehensive Assessment, Development and Assessment of Assessment Recommendation (GRADE) approach. We intend to present the findings through narrative descriptions and, if possible, through meta-analytic statistics.

**Trial registration: PROSPERO registration number**. CRD42021288387.

## Introduction

Musculoskeletal disorders are the most prevalent conditions that require rehabilitation care worldwide [1]. More than 150 clinical conditions that affect the locomotor system of individuals constitute the set of pathologies related to the musculoskeletal system (e.g., back and neck pain, osteoarthritis, regional and widespread pain disorders) [2]. They range from acute conditions with a sudden onset and short duration (e.g., fractures, sprains, and strains), to chronic disorders associated with ongoing functional limitations and disabilities [2,3]. Some studies have shown that individuals with musculoskeletal disorders have more pain, functional disability, mobility impairments and a greater risk of injuries related to falls than those without these conditions [2,4,5]. Moreover, it is estimated that 1.71 billion people have some disorder related to the musculoskeletal system in the world, contributing with 149 million years lived with disability (YLDs) [1].

A therapeutic intervention that has been applied to treat patients with musculoskeletal conditions is neural mobilization (NM), which has shown positive effects in reducing pain and improving functioning [6–9]. NM consists of combinations of joint movements that promote the gliding or the tensioning of the neural tissue and that can be performed both passively by the health professional or actively by the individual [10–13]. It is believed to facilitate the nerve gliding in relation to adjacent tissues, to facilitate neural vascularity, and to improve the axoplasmic flow, which in turn results in improved neural functioning and, consequently, in improved motor and sensory function [14] and, particularly decreased pain [15].

Previous systematic reviews varied in their aim and in their conclusions. The earliest systematic review concluded that there was only weak evidence to support the use of NM for nervous system-related pathologies [14]. More recently, Su & Lim (2016) found that NM was superior only to the minimal intervention for pain relief and disability reduction on people with chronic musculoskeletal pain, but when compared to other forms of intervention, there was no evidence that NM has a superior effectiveness in reducing pain and disability [16]. Neto et al. (2017) concluded that NM had large positive effects on pain and disability in people with low back pain and moderate effects on flexibility in healthy participants [17]. A more recent systematic review suggest that NM is effective for back and neck pain but remains unclear for other conditions [18].Our planned systematic review adds to previous reviews by updating the synthesis with recent studies, using a meta-analysis, exploring the effects of dose and type of neural mobilization on treatment response, and by broadening the outcomes of interest.

Therefore, the primary objective of our upcoming systematic review will be to synthetize the findings of studies that address the effectiveness of NM techniques on pain intensity, perceived functioning, and physical performance (e.g., flexibility, balance, and muscular strength) in adults and older adults with musculoskeletal pain. The secondary objectives will be to compare the effectiveness of different types and doses of NM and to assess the effectiveness of NM in immune responses, sensory acuity, intraneural edema and morphological and functional changes in peripheral nerves (e.g., nerve stiffness, nerve elasticity).

Based on the results of this systematic review, we intend to provide consumers and other stakeholders (e.g., patients and clinicians) with up-to-date, relevant, and high-quality information on the use of NM techniques in individuals aged 18 years and older with musculoskeletal pain providing a more precise estimate of effect of this intervention. In addition, we plan to identify specific limitations and gaps in existing scientific knowledge that merit further research.

## Materials and methods

This protocol is a document of planned methods, results and analyzes for an upcoming systematic review. It was developed considering the set of standards established by the Preferred Reporting Items for Systematic Reviews and Meta-Analysis for protocols (PRISMA-P) guidelines (S1 Checklist) [19,20]. We guarantee that any changes to the original protocol version will be documented and justified in a table summarizing protocol amendments, as well as in a specific section in the next systematic review ("Differences between the protocol and the review") with a description of the changes and its respective dates by the corresponding author (FB). The completed systematic review will be in line with PRISMA 2020 guidelines [21,22].

### Eligibility criteria

#### Study design

Randomized controlled human trials, including cluster and quasi-randomized trials. Crossover trials, as well as case series, and case reports will be excluded.

#### Participants

Adults and older adults (18 years and older), with any musculoskeletal pain condition in any time course: acute (< 6 weeks), sub-acute (< 3 months) or chronic (> 3 months) [23]. Regarding chronic musculoskeletal pain, it is defined as: (1) chronic pain emerging from musculoskeletal structures such as muscles, bones and joints related to known pathological conditions, including diseases of the nervous system; (2) and chronic pain that cannot be attributed to any underlying pathology, and therefore is considered as a condition in itself [24]. Thus, studies involving participants with clinical signs or a diagnosis of tumor/cancer, infection diseases, severe depression or other psychiatric disorders, and other systemic pathologies will be excluded.

#### Interventions

Eligible studies will have to include any form of neural mobilization (NM) techniques (i.e., sliding or tensioning performed actively or passively) as a treatment modality based on the principles proposed by Elvey, Butler and Shacklock [10,12,13] administered either as a standalone intervention or in combination with other treatment modalities (e.g., exercise, electrotherapy, education).

#### Comparators

Considering the high variability of control interventions previously identified in the literature, multiple comparisons will be considered, including non-pharmacological and pharmacological interventions as well as surgical procedures. Thus, the aim is not to conclude whether NM is more effective than a specific intervention, but rather its overall effectiveness. Studies that compare NM against a control condition (no intervention or placebo) or any intervention (including pharmacological and surgical interventions) will be considered. Also, trials comparing one NM technique with another will be included.

#### Outcomes

The main outcomes of interest will be pain intensity (e.g., Visual Analogue Scale [VAS] or Numeric Pain Rating Scale [NPRS]), physical performance (e.g., flexibility, balance, and muscular strength) measured by performance tests, and/or perceived functioning measured by self-reported questionnaires (e.g., Roland Morris Disability Questionnaire, Oswestry Disability Index, WHODAS 2.0, etc.). The secondary outcomes of interest will be data related to immune responses, sensory acuity, morphological and functional changes in peripheral nerves (e.g., nerve stiffness, nerve elasticity), and neurophysiological changes (e.g., intraneural edema and changes in temporal summation). Studies will be included if at least one of the outcomes of interest is measured.

#### Timing and setting

There will be no restrictions for follow-up time or type of setting.

#### Language

We will include articles published in English, Portuguese, or Spanish. A list of titles identified as possibly being relevant in other languages will be included in the supplementary material.

The PICO strategy is summarized in Table 1.

**Table 1:** PICO strategy.

**P (Population)**	Adults and older adults with musculoskeletal pain
**I (Intervention)**	Neural mobilization techniques
**C (Comparator)**	Inactive control intervention: • *Placebo* • *No treatment* • *Standard care* • *Waiting list control* Active control intervention: • *Different variant of neural mobilization* • *Non-pharmacologic interventions* • *Pharmacologic interventions* • *Surgical procedures*
**O (Outcomes)**	Primary outcomes: • *Pain intensity* • *Perceived functioning* • *Physical performance (muscle strength*, *flexibility*, *balance)*

### Information sources and search strategy

The search for scientific articles to be included in the review will be conducted by one researcher (FB) in the following electronic databases: Web of Science (five collections included–Web of Science Core Collection, Korean Journal Database, MEDLINE^®^, Russian Science Citation Index and SciELO Citation Index), PubMed, MEDLINE (via PubMed and Web of Science), Cumulative Index to Nursing and Allied Health Literature (CINAHL Plus with Full Text), Cochrane Central Register of Controlled Trials, Scopus, and Physiotherapy Evidence Database (PEDro). The CINAHL Plus and Cochrane Central will be accessed through EBSCO host Web. In addition to these databases, it is also intended to search in grey literature, namely unpublished sources of evidence (theses and dissertations) in the Open Access Scientific Repositories of Portugal (RCAAP–acronym in Portuguese). The International Clinical Trials Registry Platform of the World Health Organization (https://www.who.int/clinical-trials-registry-platform) and ClinicalTrials.gov will also be consulted for ongoing or recently completed trials. The search will be conducted to include articles from January 1996 to October 2021, considering that the oldest article included in previous reviews is from 1996 [14,18]. This search will be complemented by manually detecting references from bibliography of the included studies and previous reviews.

For PubMed and MEDLINE (via Web of Science), medical subject headings (MeSH) will be used and for all databases text words related to the I (intervention) and O (outcomes) dimensions of PICO strategy will be applied. All search terms have been chosen considering the PICO strategy and are all listed in S1 Appendix for each database, with the respective planned limits.

To avoid any loss of studies and considering the broad universe of musculoskeletal pain conditions and the great variability regarding control interventions identified previously in the literature, it was decided not to specify any input terms for the P (population) and C (comparator) dimensions of the PICO strategy. This will allow for a comprehensive summary of the evidence and the opportunity to explore the consistency of results across different intervention implementations.

All the studies found will be uploaded by the researcher (FB) to Mendeley Reference Manager Software [25], and to Covidence systematic review software (Veritas Health Innovation, Melbourne, Australia) to facilitate collaboration between reviewers throughout the study selection process. The researcher will remove the duplicate articles through automation tools. From Covidence platform, two reviewers (FB and another researcher that will be invited for this task) will independently screen the title and abstract of all studies and decide which ones they consider relevant for inclusion. After that, they will check the agreements and disagreements, and in a consensus meeting they will give the final decision. Then, they will read the full manuscripts and will gave their recommendation for inclusion. Any disagreements that occur at this stage will be also resolved by a consensus meeting or, when necessary, by a third researcher (AGS). Reasons for excluding trials will be recorded.

**Fig 1:**
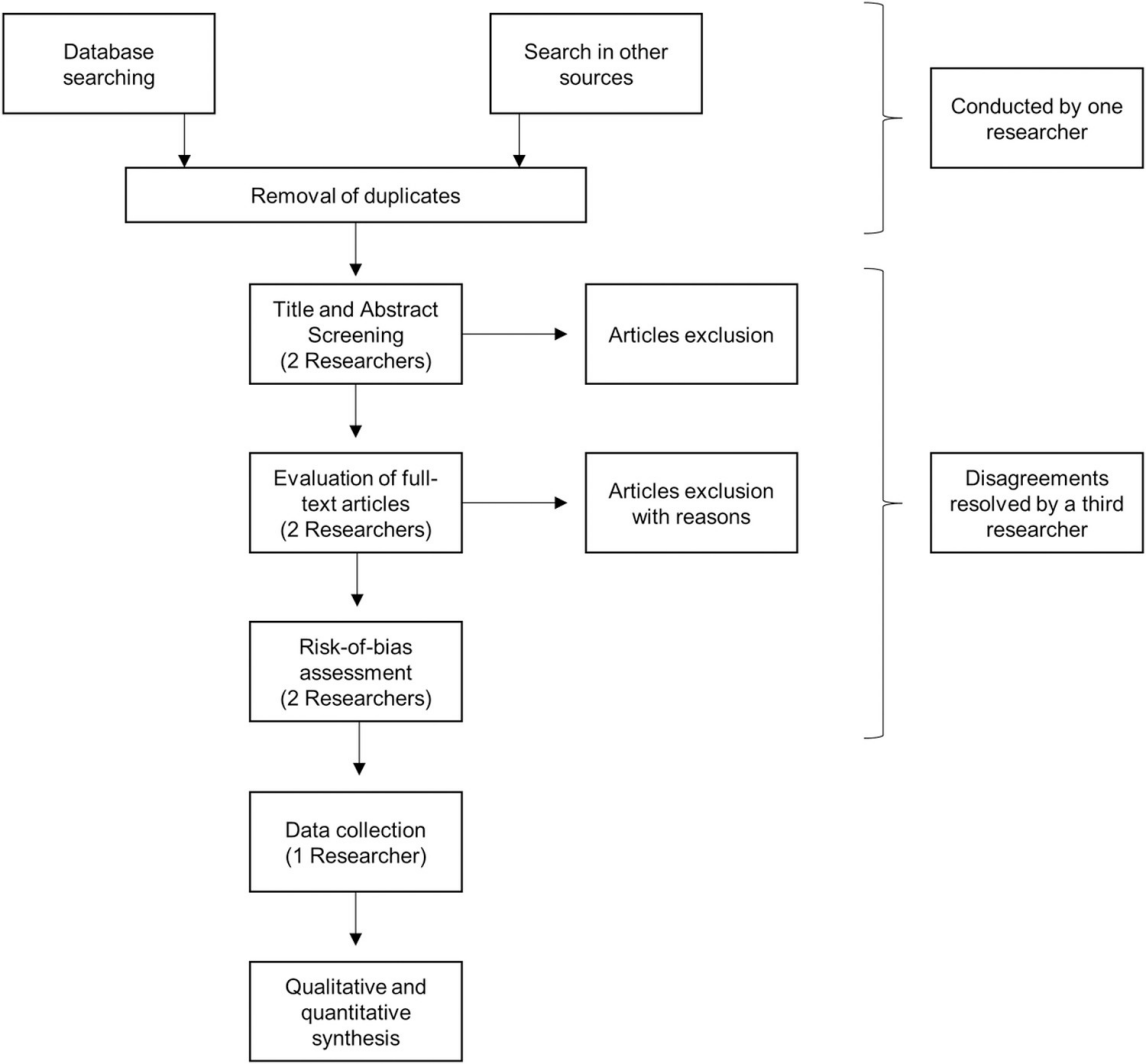
Flow diagram adapted from the PRISMA statement.

The entire screening process will be specified in a PRISMA flowchart. An adaptation of this flow diagram is shown in Fig 1, summarizing the planned study process for the systematic review.

### Risk-of-bias assessment for specific outcomes

Methodological assessment of the trials will be independently performed by two reviewers (FB and the same researcher that will screen the articles) using the Revised Cochrane risk-of-bias tool for randomized trials (RoB 2) [26,27].

RoB 2 considers, for each outcome within individual randomized trials, five domains: the randomization process, deviation from intended interventions, missing outcome data, outcome measurement, and selection of reported outcomes. The domain-level judgements lead to an overall judgement about risk-of-bias, considering that the overall judgement is the worst judgement for any domain. In the context of this study, the effect of adhering to intervention as described in the trial protocol (the “per-protocol effect”) will be considered as the effect of interest.

The possible risk-of-bias judgements are: (1) low risk-of-bias; (2) some concerns; and (3) high risk-of-bias [26,27]. Disagreements between reviewer´s assessments will be discussed and resolved in a consensus meeting between the two reviewers or, when necessary, by a third researcher (EC). Reliability of inter-rater agreement will be determined using a non-weighted kappa statistics [28]. A prior study will be carried out with a random sample of selected articles to determine the inter-rater reliability (IRR). For the purposes of performing this analysis, the answers "Yes" and "Probably yes" as well as "No" and "Probably no" of the instrument’s signaling questions will be treated as the same response [26].

To visualize the risk-of-bias assessments, "traffic light" plots of the domain-level judgments for each individual outcome and weighted bar plots of the distribution of risk-of-bias judgments within each bias domain will be drawn using the *robvis* web app [29]. Besides that, the risk-of-bias findings will be incorporated into the review through a forest plot stratified by overall risk-of-bias for each outcome in the summary findings. In a supplementary document, the answers, free text supports, and judgments of each evaluator will be presented separately for total transparency of the process.

In case of inclusion of cluster or quasi-randomized trials, RoB 2 for cluster-randomized trials (RoB 2 CRT) and ROBINS-I tool (risk of bias in non-randomized studies–Interventions) will be used, respectively [30,31].

### Data extraction and management

Data collection will be performed by one author (FB) with verification by another researcher (AGS) to reduce errors in data extraction. Qualitative and quantitative summaries will be extracted from individual studies using data extraction forms that will be developed for this purpose. Considering that during the data extraction process we may find more than one report from the same study, we will verify at this stage the juxtaposition of some information between trials (e.g., authors’ names, trial registration numbers, location and setting, specific details of the interventions, treatment comparisons, sample sizes, outcomes) to avoid data overlapping [32]. If there is more than one publication referring to the same trial, we will link them together. When discrepancies between them are very large, FB and AGS will decide which report contains the most valuable information to use as a source for the study results [32].

Data collected will include bibliographic information (authors and year of publication), patient subgroups (musculoskeletal condition), global and specific sociodemographic characteristics of each group of participants (mean age, gender ratio and mean duration of symptoms), intervention details (type of neural mobilization, modes of application, number and duration of treatment sessions, duration of follow-up, and other aspects related to dosimetry), characteristics of the control intervention, outcomes measures and their measuring instruments, time period of assessment (e.g., post-intervention, 3-months follow up) and main results for each outcome of interest.

Regarding the outcomes of interest, for each individual trial, the following data will be collected globally and from each group:

- For quantitative variables–pre- and post-intervention means and corresponding standard deviations (SD) and the standard deviation of the mean differences between pre- and post-intervention measurements. When the standard deviation of the mean difference for each group is not available, it will be calculated from the following formula [33]:

$$SDdifference=\sqrt{{SD}^{2}baseline+{SD}^{2}final-(2\times corr\times SDbaseline\times SDfinal)}$$

For quantitative variables described through order statistics, the means and standard deviation values will be estimated using the method described by Hozo et al. (2005) [34].

- For qualitative variables–pre- and post-intervention counts and frequencies;

In case of missing data, authors will be contacted by email a maximum of 3 times in a 2-month period to clarify miss information.

In studies with multiple treatment groups, we will collect data from each group separately, so that it is possible to make distinct comparisons between the treatment arms and the control group. When there are trials with evaluations for more than one body region, data from all of them will be collected. Furthermore, in studies with many evaluation moments, data from all time points will be collected and pooled for analyzes (e.g., short term [<5 sessions], medium term [5 to 10 sessions], and long term [>10 sessions]).

Qualitative and quantitative data, as information from risk-of-bias assessments, will be summarized in Summary of Findings tables in accordance with Cochrane guidelines [35].

### Data synthesis

Depending on the number of trials included, how different studies measure the variables, and the diversity in the methods of conducting the trials, it will be possible to yield a quantitative summary of the results pooling data with meta-analytical techniques using R software environment for statistical computing and graphics [36].

Considering the possibility of performing meta-analyses, studies will be pooled regarding specific outcomes and musculoskeletal condition to generate estimated effect sizes for each of the outcomes of interest within each musculoskeletal pathology.

Regarding the meta-analysis itself, the effect sizes and their 95% CI will be determined and combined by the standardized mean differences (SMDs) for quantitative variables, considering that in previous research many different outcome measures were identified between trials. Based on a previous knowledge about the different characteristics between studies involving NM techniques in previous reviews [14,16–18], we will consider a random effects model in possible future meta-analyses.

Statistical heterogeneity will be investigated using Cochran’s Q statistic [37] for which a significant p-value (< 0.1) will be defined [38]. The I^2^ statistic will also be performed, with values of 25%, 50% and 75% reflecting low, moderate and high statistical heterogeneity, respectively [39,40]. This type of analysis assumes evaluating the proportion of dispersion observed due to real differences in effect sizes beyond sampling error.

**Fig 2:**
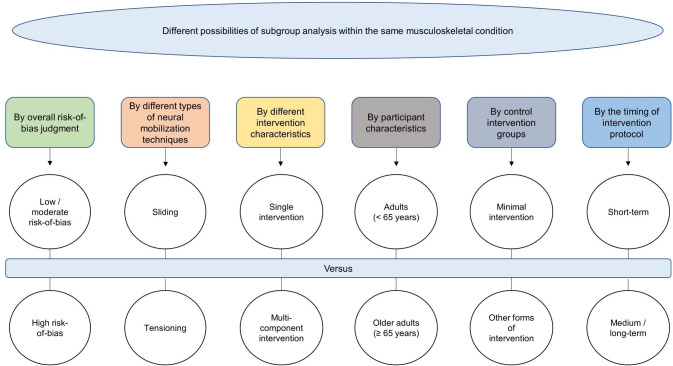
Subgroup analyses.

Heterogeneity, subgroups and meta-regression analysis will be performed to describe and surpass difficulties imposed by the lack of homogeneity between studies (e.g., stratify meta-analysis by overall risk-of-bias judgement [low or moderate risk-of-bias *versus* high risk-of-bias], by different types of neural mobilization [sliding *versus* tensioning], by different intervention characteristics [single-component intervention study or a multi-component intervention study], by participant characteristics [age], by control intervention groups [minimal intervention *versus* other forms of intervention], by the timing of intervention protocol [short term *versus* medium/long term], and meta-regression to test the impact of dose on effect size) (Fig 2).

Sensitivity, inflation bias (“p-hacking”) and publication bias statistical analyzes will be considered when justified to assess robustness of the synthetized results (e.g., funnel plots, Egger´s test, “p-curve”). We will also consider whether there is selective reporting of outcomes, checking the outcomes reported in the published trials and those established in its protocols. When no protocol is available, outcomes reported in the methods and results sections of the published study will be checked for comparison. The Outcome Reporting Bias in Trials (ORBIT) classification system will be used for this task [41].

In situations where statistical pooling is not possible, the results of the systematic review will only be presented in a narrative form, exploring the relationship and findings within and between the included trials in line with the guidance from the Economic and Social Research Council (ESRC) [42]. For that, the synthesis will be described using information presented in the text and in tables to summarize and explain the characteristics and findings of the included studies.

### Certainty of evidence

Certainty of evidence for each outcome across studies will be assessed using the Grading of Recommendations Assessment, Development and Evaluation (GRADE) comprehensive approach [43]. The final rating for certainty of evidence may be: high, moderate, low, or very low [43]. GRADEpro computer software (McMaster University, Ontario) will be used to facilitate the process of developing GRADE evidence profiles (EPs) and Summary of Findings (SoFs) tables [44,45].

The rating will be conducted independently by two review authors (FB and AGS) for each outcome considered. Any disagreement will be discussed and resolved by consensus, if necessary, with a third review author (EC).

## Discussion

The proposed systematic review intends to assess the effectiveness of applying NM techniques in comparison with other therapeutic interventions in adults and older adults with musculoskeletal pain.

In addition to pain, disability and aspects related to physical performance are also relevant variables that may be compromised in musculoskeletal disorders [2–5]. Thus, it is important to investigate therapeutic interventions aimed at improving these dysfunctions of signs and symptoms related to musculoskeletal conditions.

Although NM has shown some positive effects in treating people with musculoskeletal pain [6–9], previous systematic reviews have shown limited or inconclusive evidence to support the use of this intervention in these population. As stated before, most previous reviews have focused only on specific musculoskeletal conditions or only on limited outcomes of interest, with a narrow approach of the NM application [17,46–51], with the exception of three of them [14,16,18].

Taking this into account, we intend to develop a broad systematic review encompassing studies related to any musculoskeletal condition and developed in any setting context. This will allow to provide a comprehensive view of the real scientific evidence on the use of NM techniques in people who suffer the consequences of musculoskeletal disorders. Because of this, and considering that interpretation of results in broad systematic reviews may be difficult and evidence sparse [52], we plan narrative descriptions and statistical analyzes that can explain the high heterogeneity we expect to find.

A comprehensive summary of the scientific evidence on the effectiveness of using NM in the treatment of musculoskeletal pain is crucial for the development of protocols involving this specific intervention, as well as to allow the design of future methodological strategies in conducting randomized controlled trials that make it possible to respond to the research gaps that still exist.

Considering the high variability in the application of NM techniques (e.g., passive *versus* active approach, sliding *versus* tensioning techniques, global *versus* local tissue mobilization), systematic assessment of aspects of this intervention in existing trials could contribute valuable insights into which neural mobilization works best to relieve pain, improve functioning, and physical performance characteristics in people with musculoskeletal pain.

## Supporting information

S1 Checklist PRISMA-P checklist. (DOC)

S1 Appendix Search strategy. (XLSX)
