# Supplementary material for: Effectiveness of neural mobilization on pain intensity, disability, and physical performance in adults with musculoskeletal pain—A protocol for a systematic review of randomized and quasi-randomized controlled trials and planned meta-analysis
Source: PLoS One. 2022 Mar 10;17(3):e0264230. doi: 10.1371/journal.pone.0264230 (PMC8912212; doi:10.1371/journal.pone.0264230)
Supplement: S1 Checklist — (DOC) [file pone.0264230.s001.doc]

**PRISMA-P (Preferred Reporting Items for Systematic review and Meta-Analysis Protocols) 2015 checklist: recommended items to address in a systematic review protocol***

| Section and topic | Item No | Checklist item |
| --- | --- | --- |
| ADMINISTRATIVE INFORMATION | | |
| Title: |  |  |
| Identification | 1a | Identify the report as a protocol of a systematic review PAGE 1, LINE 2 |
| Update | 1b | If the protocol is for an update of a previous systematic review, identify as such Not Applicable |
| Registration | 2 | If registered, provide the name of the registry (such as PROSPERO) and registration number  *Following the PROSPERO database guidelines, we will register the protocol after the peer review is complete to ensure that no significant changes will be made after registration.* |
| Authors: |  |  |
| Contact | 3a | Provide name, institutional affiliation, e-mail address of all protocol authors; provide physical mailing address of corresponding author PAGE 1, LINES 6, 8 – 13, 15 |
| Contributions | 3b | Describe contributions of protocol authors and identify the guarantor of the review PAGE 1, LINES 16, 17; PAGE 15, LINES 362 – 371 |
| Amendments | 4 | If the protocol represents an amendment of a previously completed or published protocol, identify as such and list changes; otherwise, state plan for documenting important protocol amendments Not Applicable |
| Support: |  |  |
| Sources | 5a | Indicate sources of financial or other support for the review Not Applicable |
| Sponsor | 5b | Provide name for the review funder and/or sponsor Not Applicable |
| Role of sponsor or funder | 5c | Describe roles of funder(s), sponsor(s), and/or institution(s), if any, in developing the protocol Not Applicable |
| INTRODUCTION | | |
| Rationale | 6 | Describe the rationale for the review in the context of what is already known PAGES 3 – 5, LINES 64 – 107 |
| Objectives | 7 | Provide an explicit statement of the question(s) the review will address with reference to participants, interventions, comparators, and outcomes (PICO) PAGE 4, LINES 95 – 101 |
| METHODS | | |
| Eligibility criteria | 8 | Specify the study characteristics (such as PICO, study design, setting, time frame) and report characteristics (such as years considered, language, publication status) to be used as criteria for eligibility for the review PAGES 5 – 7, LINES 118 – 166 |
| Information sources | 9 | Describe all intended information sources (such as electronic databases, contact with study authors, trial registers or other grey literature sources) with planned dates of coverage PAGES 7,8, LINES 167 – 182 |
| Search strategy | 10 | Present draft of search strategy to be used for at least one electronic database, including planned limits, such that it could be repeated PAGE 8, LINES 183 – 192, APPENDIX 1 |
| Study records: |  |  |
| Data management | 11a | Describe the mechanism(s) that will be used to manage records and data throughout the review PAGE 8, LINES 183 – 203 |
| Selection process | 11b | State the process that will be used for selecting studies (such as two independent reviewers) through each phase of the review (that is, screening, eligibility, and inclusion in meta-analysis) PAGE 8, LINES 196 – 203 |
| Data collection process | 11c | Describe planned method of extracting data from reports (such as piloting forms, done independently, in duplicate), any processes for obtaining and confirming data from investigators PAGE 10, LINES 240 – 249 |
| Data items | 12 | List and define all variables for which data will be sought (such as PICO items, funding sources), any pre-planned data assumptions and simplifications PAGE 6, LINES 149 – 157; PAGES 10 – 12, LINES 250 – 279 |
| Outcomes and prioritization | 13 | List and define all outcomes for which data will be sought, including prioritization of main and additional outcomes, with rationale PAGE 6, LINES 149 – 157 |
| Risk of bias in individual studies | 14 | Describe anticipated methods for assessing risk of bias of individual studies, including whether this will be done at the outcome or study level, or both; state how this information will be used in data synthesis PAGES 9, 10, LINES 209 – 237 |
| Data synthesis | 15a | Describe criteria under which study data will be quantitatively synthesised PAGE 12, LINES 282 – 285 |
|  | 15b | If data are appropriate for quantitative synthesis, describe planned summary measures, methods of handling data and methods of combining data from studies, including any planned exploration of consistency (such as I^2^, Kendall’s τ) PAGE 12, LINES 281 – 299 |
|  | 15c | Describe any proposed additional analyses (such as sensitivity or subgroup analyses, meta-regression) PAGES 12, 13, LINES 300 – 314 |
|  | 15d | If quantitative synthesis is not appropriate, describe the type of summary planned PAGE 13, LINES 315 – 319 |
| Meta-bias(es) | 16 | Specify any planned assessment of meta-bias(es) (such as publication bias across studies, selective reporting within studies) PAGE 13, LINES 308 – 314 |
| Confidence in cumulative evidence | 17 | Describe how the strength of the body of evidence will be assessed (such as GRADE) PAGES 13, 14, LINES 321 – 330 |

*** It is strongly recommended that this checklist be read in conjunction with the PRISMA-P Explanation and Elaboration (cite when available) for important clarification on the items. Amendments to a review protocol should be tracked and dated. The copyright for PRISMA-P (including checklist) is held by the PRISMA-P Group and is distributed under a Creative Commons Attribution Licence 4.0.**

*From: Shamseer L, Moher D, Clarke M, Ghersi D, Liberati A, Petticrew M, Shekelle P, Stewart L, PRISMA-P Group. Preferred reporting items for systematic review and meta-analysis protocols (PRISMA-P) 2015: elaboration and explanation. BMJ. 2015 Jan 2;349(jan02 1):g7647.*
